# Prevalence of and Sex-Related Differences in Excessive Daytime Sleepiness in Adolescents and Young Adults with Malocclusion

**DOI:** 10.3390/diagnostics15121527

**Published:** 2025-06-16

**Authors:** Shuzo Sakata, Ryo Kunimatsu, Kazutaka Ikeda, Katsuhito Sugai, Shintaro Ogashira, Kotaro Tanimoto

**Affiliations:** 1 Department of Orthodontics, Division of Oral Health and Development, Hiroshima University Hospital, Hiroshima 734-8553, Japan; shuzosakata@hiroshima-u.ac.jp (S.S.); kazutaka-ikeda@hiroshima-u.ac.jp (K.I.); katu1118@hiroshima-u.ac.jp (K.S.); milk595@hiroshima-u.ac.jp (S.O.); 2 Department of Orthodontics and Craniofacial Developmental Biology, Graduate School of Biomedical and Health Sciences, Hiroshima University, Hiroshima 734-8553, Japan; tkotaro@hiroshima-u.ac.jp

**Keywords:** daytime sleepiness, Epworth Sleepiness Scale, malocclusion

## Abstract

**Background/Objectives**: Excessive daytime sleepiness (EDS) has been suggested to negatively affect academic performance and behavior. Malocclusion is reportedly a risk factor for sleep-disordered breathing and may be associated with daytime sleepiness. This study investigated the age and sex of patients with malocclusion who are at increased risk of EDS and collected data for future EDS screening. **Methods**: We analyzed 556 patients with malocclusion aged 6–29 years to identify age- and sex-specific differences in the risk of EDS. Adults were surveyed using the Epworth Sleepiness Scale (ESS), and non-adults were surveyed using the Epworth Sleepiness Scale for Children and Adolescents. **Results**: The mean ESS score was 4.1 ± 4.1, and the median score was 3. The mean score tended to increase significantly with age. In adolescents, ESS scores increased gradually. In adolescents and young adults, ESS scores were significantly higher in females than in males, and the prevalence of EDS in young adults was 16.7% in men and 32.1% in women, with a marked sex-related difference. **Conclusions**: In patients with malocclusion, daytime sleepiness may gradually increase from adolescence to young adulthood and be more pronounced in females. The prevalence of EDS in young adults seems higher in women than in men. Our findings could aid in the early identification of EDS and facilitate multidisciplinary cooperation between dentists and sleep specialists. Early identification of sleep problems and prompt intervention during the high-risk period for developing EDS could improve the quality of life for many adolescents and contribute to improved public health.

## 1. Introduction

Excessive daytime sleepiness (EDS) is widely recognized as a serious societal problem. Among adults, it is associated with an increased risk of traffic accidents, poor daytime performance, and reduced quality of life (QOL) [1,2]. Most EDS cases in adults are associated with sleep deprivation and sleep-disordered breathing, including obstructive sleep apnea; however, insomnia and other sleep disorders can also cause EDS [3]. On the other hand, pediatric EDS has become a significant international concern in recent years [4]. Pediatric EDS is related to physiological changes in sleep architecture associated with growth, hormonal balance changes during puberty, and life schedule changes associated with schooling, as well as an increased risk of sleep-disordered breathing due to adenoids and enlarged palatine tonsils and sleep disorders such as narcolepsy, idiopathic hypersomnia, and Kleine–Levin syndrome; thus, the etiology is greatly complex [5,6]. It may also be associated with poor academic performance, low motivation, and defiant behavior [7]. Therefore, investigating EDS will help teachers and parents to better understand these issues of children with EDS. Furthermore, it will help clinicians to perform appropriate diagnostic screening and early intervention to prevent pediatric sleep disorders related to EDS.

Malocclusion occurs in approximately half of the population and is considered a major oral disease, along with dental caries and periodontal diseases [8,9]. Incongruent maxillofacial morphology, one of the causes of malocclusion, may be a risk factor for sleep-disordered breathing in children and adults, while incorrect breathing habits have been suggested to affect maxillofacial growth [10,11]. Thus, a close relationship may exist between malocclusion and sleep problems in children and adults. Furthermore, several reports have suggested that improving malocclusion in children effectively improves sleep-disordered breathing [12,13]. In other words, the presence of malocclusion is likely to be closely related to the presence of sleep-disordered breathing.

Although it is widely known that sleep-disordered breathing can cause excessive daytime sleepiness, reports of a direct association between malocclusion and EDS are limited. A recent study showed a higher prevalence of EDS in children with malocclusion than in children with normal occlusion [14]. However, this study lacked detailed subgrouping based on patient background, and further detailed investigation is needed to understand the causes of EDS. In particular, it would be desirable to obtain data on EDS from populations stratified by age and sex, given that sleep is strongly influenced by age and sex. Therefore, the aim of this study was to investigate the prevalence of EDS and its association with age and sex in a group of patients with malocclusion who visited a hospital for orthodontic treatment. EDS is a social problem that significantly reduces patients’ quality of life, and early identification and appropriate intervention are important. To this end, it is necessary to examine in detail the factors that increase the risk of EDS. The findings of this study will promote multidisciplinary collaboration by enabling dentists who identify patients at high risk for EDS to refer them to sleep specialists at an early stage. In addition, the early prevention of potential EDS through early intervention could effectively contribute to improved public health.

## 2. Materials and Methods

### 2.1. Study Approval, Criteria, Setting, and Participants

This study was approved by the Ethics Review Committee of Hiroshima University (Approval No. E2015-0056). The data were obtained from patients with malocclusion aged 6–29 years who visited the Orthodontics Department of Hiroshima University Hospital and were scheduled to undergo orthodontic treatment. The target age range was selected to encompass the stages of sleep development from childhood to adulthood and to cover the period when the clinical manifestations of malocclusion and the indications for treatment are important. All eligible patients or their parents were administered a questionnaire as part of the routine initial orthodontic examination. Questionnaires were completed at home and were collected by the treating orthodontist. Exclusion criteria included a history of diseases associated with dental and craniofacial abnormalities (e.g., cleft lip and palate, Down syndrome, craniosynostosis, Prader–Willi syndrome, Larsen syndrome, Pierre Robin sequence, Noonan syndrome, chromosomal deletion syndrome, Klippel–Trénaunay–Weber syndrome, Marfan syndrome, osteogenesis imperfecta, hemifacial microsomia, Léri–Weill dyschondrosteosis, Gorlin syndrome, oculodentodigital syndrome, Turner syndrome, CHARGE syndrome, achondroplasia, neurofibromatosis, congenital myopathy, muscular dystrophy, etc.), and a history of developmental disability (e.g., attention-deficit hyperactivity disorder, autism spectrum disorder, mental retardation, etc.).

### 2.2. Assessments

To assess subjective daytime sleepiness in adult patients, we used the Japanese version of the Epworth Sleepiness Scale (JESS) [15], a revision of the Epworth Sleepiness Scale (ESS) [16] for Japanese patients. The ESS is used worldwide to assess how drowsy a person feels in various situations where he or she may become aware of drowsiness during the daytime. The questionnaire consists of eight items, each with scores ranging from 0 to 3. The maximum total ESS score is 24. The higher the total score, the more likely the patients are to feel sleepy during the daytime. For pediatric patients, the Epworth Sleepiness Scale for Children and Adolescents (ESS-CHAD) [17] was used. This is a modification of the ESS questionnaire that excludes situations that children and adolescents are less likely to encounter. For example, the entry regarding alcohol is removed, and “participation in meetings” is changed to “participation in classes”. Further, the phrasing of question 8 has been changed to “while sitting and eating a meal”. Patients who obtained a score > 10 on either the ESS or ESS-CHAD assessments were determined to have EDS. The mean ESS score and prevalence of EDS in patients with malocclusion were grouped and investigated according to age and sex.

### 2.3. Statistical Analysis

The minimum required sample size was determined using G*Power software ver. 3.1.9.7. Based on an effect size of 0.5, a significance level of 0.05, and a power of 0.8, the required sample size was estimated to be 140. All statistical analyses were performed using Bell Curve for Excel ver. 3.00 (Social Survey Research Information), with a significance level of *p* < 0.05. Data were first assessed for normality using the Shapiro–Wilk test, and since normality was rejected, nonparametric methods were used for subsequent tests. Age-related changes in mean ESS scores were analyzed for trends using the Jonckheere–Terpstra test. Multiple comparisons of ESS scores at each stage of adolescence were performed using the Steel–Dwass test. Comparisons between the two groups by sex were performed using the Mann–Whitney U test for continuous variables and Fisher’s exact test for categorical variables.

## 3. Results

**Figure 1:**
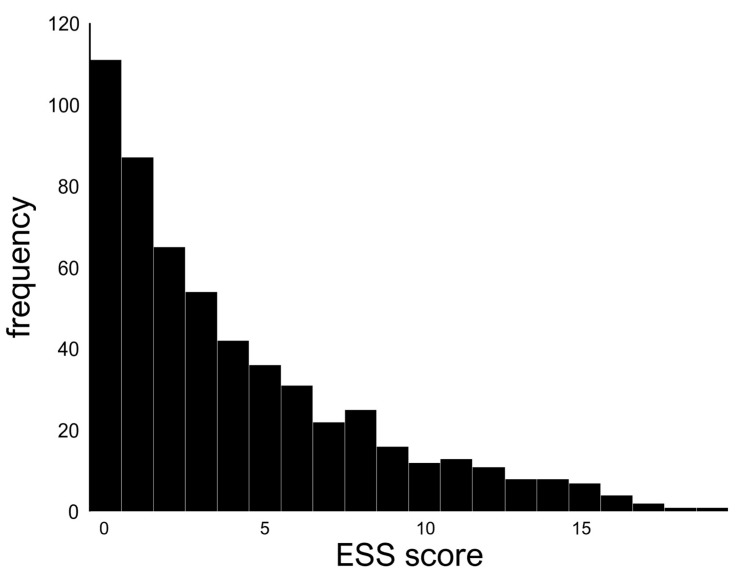
Distribution of ESS scores in all patients. The y-axis represents the number of patients.

**Figure 2:**
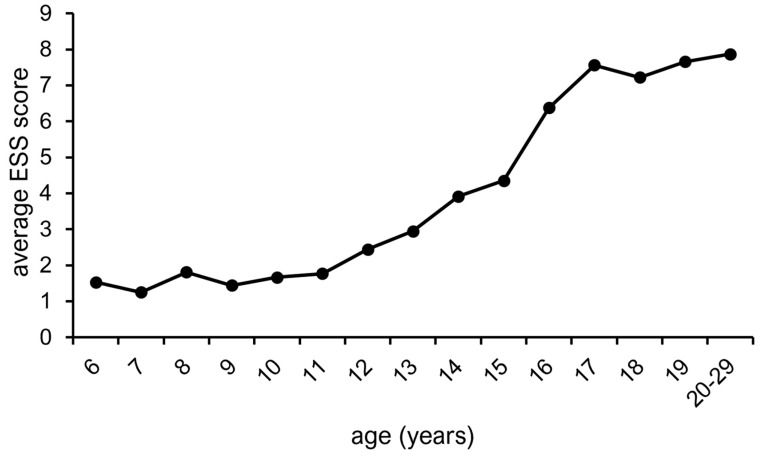
Age-related changes in the mean ESS score.

In total, 556 patients were included (males, *n* = 215 [38.7%]; females, *n* = 341 [61.3%]). The mean age was 14.1 ± 6.1 years, and 238 (42.8%) patients were ≤11 years old, 155 (27.9%) were 12–17 years old, and 163 (29.3%) were ≥18 years old. The data distribution of the ESS scores of the samples is shown in Figure 1. The histograms did not show a normal distribution. The skewness and kurtosis were 1.2 and 0.8, respectively, and no outliers were observed. The most frequent ESS score was 0 (*n* = 111), and the highest ESS score was 19. The frequency gradually decreased as the score increased. The median ESS score was 3, and the mean ESS score was 4.1 ± 4.1. The mean ESS scores increased significantly with age (Figure 2). From 6 to 11 years of age, the mean ESS score remained at <2. However, the ESS score began to increase gradually from age 12 onward, with the largest increase in the mean score (i.e., 2) occurring between ages 15 and 16 years. At the age of 17 years, the mean score exceeded 7, with no remarkable increase in the score at older ages.

**Figure 3:**
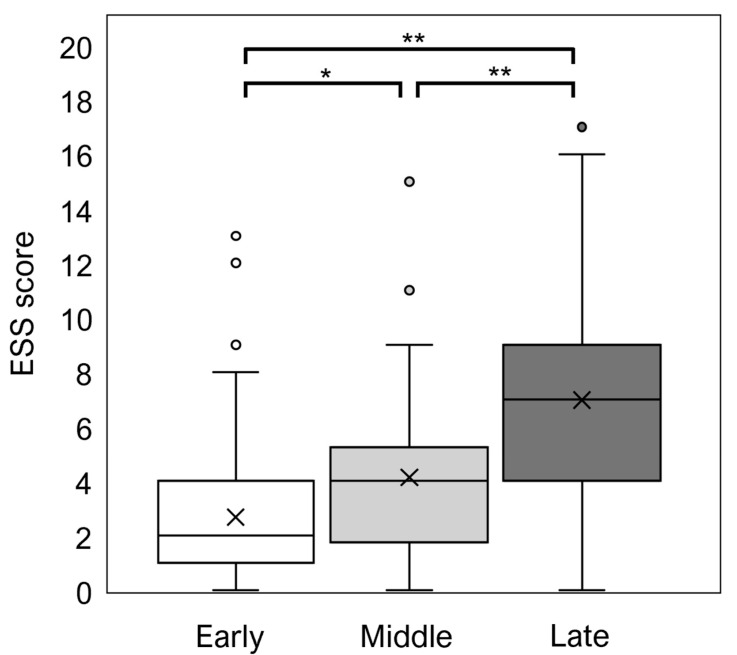
Comparison of ESS scores at early (12–13 years), middle (14–15 years), and late (16–17 years) stages of adolescence. Boxes represent interquartile ranges (IQRs), horizontal lines in boxes represent median values, whiskers represent minimum and maximum values, ✕ represents the mean value, and dots represent outliers. * *p* < 0.05; ** *p* < 0.01 (Steel–Dwass test).

**Figure 4:**
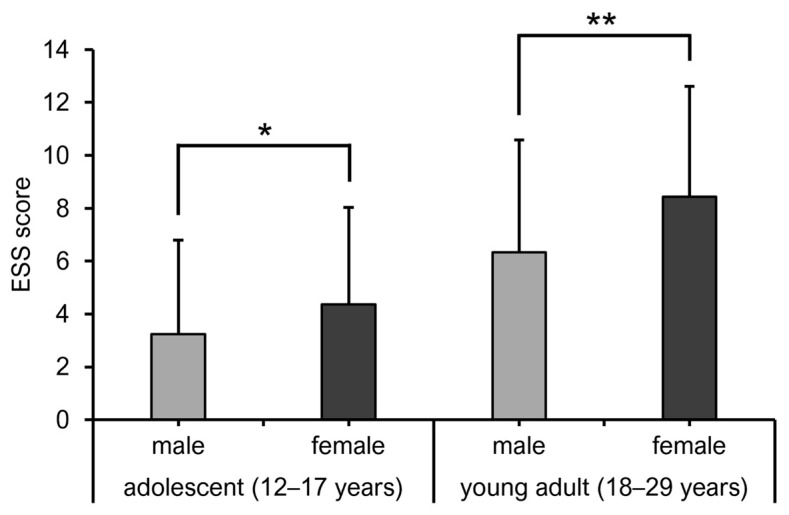
Comparison of ESS scores of males and females. * *p* < 0.05; ** *p* < 0.01 (Mann–Whitney U test).

**Figure 5:**
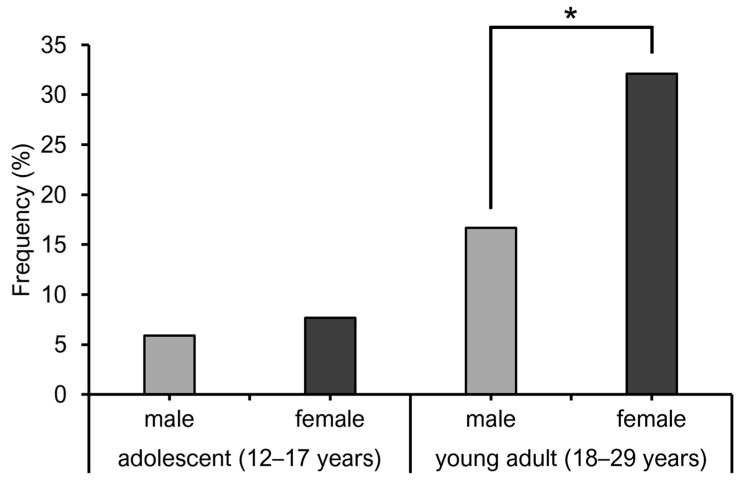
Comparison of EDS prevalence rates among males and females. * *p* < 0.05 (Fisher’s exact test).

When adolescence (12–17 years) was divided into three stages (early, middle, and late) at 2-year intervals, the mean ESS scores for the early, middle, and late stages were 2.7, 4.1, and 7, respectively, indicating a significant increase in ESS scores from the early to late stages (Figure 3). ESS scores of adolescents (age, 12–17 years) and young adults (age, 18–29 years) by sex are shown in Figure 4. Females had significantly higher ESS scores than males among both adolescents and young adults. In adolescents, the prevalence rate of EDS was 5.9% in males and 7.7% in females, with no significant sex-related difference (Figure 5). However, in young adults, the EDS prevalence rate was 16.7% in men and 32.1% in women, indicating a significant difference.

## 4. Discussion

Daytime sleepiness is associated with sleep debt or circadian rhythm changes [18]. A strong daytime sleep pressure, known as EDS, negatively affects academic and social activities [19]. Therefore, in this study, we investigated the prevalence of EDS and changes in daytime sleepiness in children and young adults with malocclusion.

An ESS score of 10 or less is considered to indicate the absence of EDS; however, patients with a score of 0 or 10 have different risks of developing EDS [20]. Therefore, we investigated the changes in the mean ESS score with age to estimate the changes in the risk of developing EDS at each age. The results showed that ESS scores increased with age, notably between 15 and 16 years. The period from 12 to 17 years of age was also identified as a time of gradual increase in ESS scores. Changes in social schedules associated with schooling have been reported to contribute to increased daytime sleepiness [21]. In Japan, the age of 16 corresponds to the first year of high school, and the change in life schedule associated with moving on from junior high school may have contributed to the increased prevalence of EDS in males and females. Exposure to electronic media and increased opportunities for socialization have also been shown to contribute to decreased sleep duration during adolescence [22]. Moreover, the prevalence of EDS has been reported to increase in both males and females during adolescence [23], and the present results are consistent with these trends. Thus, the results of the present study suggest that the risk of developing EDS increases between 12 and 17 years of age owing to various factors.

Sex-related differences in mean ESS scores showed a female predominance in both adolescents and young adults. Major factors contributing to daytime sleepiness include insufficient sleep duration [24] and poor sleep quality [25]. Many studies have also confirmed that adolescent and adult females sleep fewer hours and have poorer sleep quality than males [26,27,28]. This evidence suggests that females are at a higher risk of experiencing EDS than males. However, this may not be explained solely by the direct effects of sex hormones on sleep [29]. For example, depression, which is a risk factor for daytime sleepiness, is significantly more prevalent in females than in males after puberty [30]. Females may also be more susceptible to the adverse effects of psychological factors on sleep than males [31]. Given these reports on female vulnerability to sleep problems, the present results could reflect sleep problems among adolescent and young adult females.

In the present study, the prevalence rate of EDS in adolescents was approximately 6–7% in both males and females, which did not differ much from previous reports on EDS prevalence in children [4,29]. Therefore, the prevalence of EDS in adolescents with malocclusion may not differ considerably from the prevalence of EDS in the general adolescent population. However, various subtypes of malocclusion exist, which may or may not be associated with sleep problems [32]. Therefore, detailed studies with stratified sampling by malocclusion subtype are warranted. Regarding the prevalence of EDS in young adults, the prevalence rate remained at approximately 16% in males, whereas in females, the prevalence rate exceeded 30%. However, considering that the prevalence rate of EDS in the general population ranges from 8.5% to 22% [33] and the prevalence rate was 12.2% in males and 14.8% in females according to a study of the general Japanese adult population [34], the prevalence rate in the young adult female sample in this study was relatively high. Thus, malocclusion may be associated with EDS in young adult females; however, no definite conclusions can be drawn from the present study alone.

One of the major strengths of this study is that it covered a wide age range from children to young adults. This allowed us to estimate the age groups at high risk of developing EDS from adolescence to the young adult stage. Furthermore, we observed differences in the risk and prevalence of EDS between adolescents and young adults of both sexes. The results indicate an increased risk of EDS after adolescence and vulnerability to sleepiness in females, underscoring the importance of parent and teacher understanding and primary care by specialists. If we can identify sleep problems and intervene early during the high-risk period for developing EDS, we will be able to improve the QOL of many adolescents and contribute to improved public health.

This study has several limitations. First, because this was a cross-sectional study of a population sample, caution must be exercised when interpreting the results. Because the present results are homogeneous to variations in individual genetic and socioeconomic backgrounds, additional longitudinal studies with well-controlled confounding factors should be conducted. Second, the gold standard of assessment of daytime sleepiness is an objective evaluation using the multiple sleep latency test (MSLT) [35,36]. However, performing the MSLT on large samples is not feasible owing to limited access to research facilities and costs. The ESS and ESS-CHAD were the most suitable assessment tools for this study because of their widely accepted usefulness and adequate clinical utility and validity [37,38]. In the future, additional studies using subjective data from questionnaires and objective data from MSLTs should be added, and calibrations for subjective data should be developed. Third, because this was a single-center study of patients with malocclusion who visited an orthodontist, the results might differ in other populations. The influence of ethnicity on daytime sleepiness has been reported in many studies [39,40,41]. As most study participants were Japanese, the present results require caution regarding ethnic differences. Therefore, additional international multicenter studies with large samples from the general population and patients with malocclusion are warranted.

In patients with malocclusion, the risk of developing EDS may gradually increase during puberty, and those aged 15–16 years may be at a particularly high risk. Moreover, adolescent and young adult females may be at a higher risk of EDS than males of the same age group, and young adult females have a higher prevalence of EDS than young adult males.

## 5. Conclusions

The aim of this study was to examine the relationship between age and sex and the risk of EDS in a group of patients with malocclusion. Our results suggest that the risk of EDS gradually increases during adolescence, especially around the age of 15 to 16 years. Our findings also indicated that the risk of EDS from adolescence to young adulthood may be at higher in females than males. The results of this study suggest that early identification of patients at high risk for EDS may promote multidisciplinary collaboration between dentists and sleep specialists, which, in turn, may contribute to improved public health through early intervention for EDS.

## Data Availability

The data generated and analyzed during the current study are available from the corresponding author upon reasonable request.

## Abbreviations

The following abbreviations are used in this manuscript:

EDS	Excessive daytime sleepiness
ESS	Epworth Sleepiness Scale
ESS-CHAD	Epworth Sleepiness Scale for Children and Adolescents
JESS	Japanese version of the Epworth Sleepiness Scale
MSLT	Multiple sleep latency test
QOL	Quality of life
